# Inhibition of basal-like breast cancer growth by FTY720 in combination with epidermal growth factor receptor kinase blockade

**DOI:** 10.1186/s13058-017-0882-x

**Published:** 2017-08-04

**Authors:** Janet L. Martin, Sohel M. Julovi, Mike Z. Lin, Hasanthi C. de Silva, Frances M. Boyle, Robert C. Baxter

**Affiliations:** 1 Kolling Institute, University of Sydney, Royal North Shore Hospital, St Leonards, NSW 2065 Australia; 2 Patricia Ritchie Centre for Cancer Care and Research, Mater Hospital, North Sydney, NSW 2065 Australia; 3 Present address: Westmead Hospital, Westmead, NSW 2145 Australia

**Keywords:** Gefitinib, FTY720, IGFBP-3, Sphingosine kinase, Basal-like breast cancer

## Abstract

**Background:**

New molecular targets are needed for women with triple-negative breast cancer (TNBC). This pre-clinical study investigated the combination of the EGFR inhibitor gefitinib with the sphingosine kinase (SphK) inhibitor FTY720 (Fingolimod), aiming to block tumorigenic signaling downstream of IGFBP-3, which is abundantly expressed in basal-like TNBC.

**Methods:**

In studies of breast cancer cell growth in culture, proliferation was monitored by IncuCyte live-cell imaging, and protein abundance was determined by western blotting. In vivo studies of mammary tumor growth used two models: orthotopic xenograft tumors derived from three basal-like TNBC cell lines, grown in immune-deficient mice, and syngeneic murine 4T1 tumors grown in immune-competent mice. Protein abundance in tumor tissue was assessed by immunohistochemistry.

**Results:**

Quantitated by live-cell imaging, the inhibitor combination showed synergistic cytostatic activity in basal-like cell lines across several TNBC molecular subtypes, the synergy being decreased by IGFBP-3 downregulation. Suppression of the tumorigenic mediator CD44 by gefitinib was potentiated by FTY720, consistent with CD44 involvement in the targeted pathway. In MDA-MB-468 and HCC1806 orthotopic TNBC xenograft tumors in nude mice, the drug combination inhibited tumor growth and prolonged mouse survival, although this effect was not significant for the gefitinib-resistant cell line HCC70. Combination treatment of murine 4T1 TNBC tumors in syngeneic BALB/c mice was more effective in immune-competent than immune-deficient (nude) mice, and a relative loss of tumor CD3 (T-cell) immunoreactivity caused by FTY720 treatment alone was alleviated by the drug combination, suggesting that, even at an FTY720 dose causing relative lymphopenia, the combination is still effective in an immune-competent setting. Immunohistochemistry of xenograft tumors showed significant enhancement of caspase-3 cleavage and suppression of Ki67 and phospho-EGFR by the drug combination, but SphK1 downregulation occurred only in MDA-MB-468 tumors, so is unlikely to be integral to treatment efficacy.

**Conclusions:**

Our data indicate that targeting IGFBP-3-dependent signaling pathways through gefitinib-FTY720 co-therapy may be effective in many basal-like breast cancers, and suggest tissue IGFBP-3 and CD44 measurement as potential biomarkers of treatment efficacy.

**Electronic supplementary material:**

The online version of this article (doi:10.1186/s13058-017-0882-x) contains supplementary material, which is available to authorized users.

## Background

In approximately 15% of invasive breast cancers, estrogen receptor (ER) and progesterone receptor expression is low or undetectable, and human epidermal growth factor (EGF) receptor-2 (HER2) is not overexpressed. These triple-negative breast cancers (TNBC) carry a poorer prognosis than other breast cancer types [1, 2]. A majority of TNBC falls into the basal-like molecular classification, and conversely, most basal-like cancers are triple-negative [3, 4]. Among six defined molecular subtypes of TNBC [5], those identified as basal-like 1 (BL1), basal-like 2 (BL2), immunomodulatory (IM), and mesenchymal are overwhelmingly classified as having basal-like molecular signatures [3, 4]. Since TNBC is not susceptible to ER- or HER2-targeted therapies, these cancers are generally treated with adjuvant, and sometimes neoadjuvant, chemotherapy, often with substantial survival benefit [4]. Adjuvant radiotherapy may also improve survival in some patient groups [6]. However, the risk of relapse within the first 3–4 years is relatively high [2]. In attempts to overcome this, numerous targeted therapies have been trialed, alone or in combination with chemotherapy [4], but the need remains for new treatment options for women with TNBC.

The EGF receptor (EGFR) is often highly expressed in TNBC tumors, and is prognostic for worse disease-free survival [7], although EGFR mutation is uncommon [8]. Single-agent blockade of EGFR signaling has been disappointing in TNBC patients [9, 10], suggesting that combination therapies may be needed. The growth-regulatory protein, insulin-like growth factor binding protein-3 (IGFBP-3), potentiates EGFR signaling in MCF-10A mammary epithelial cells – which are triple-negative, basal B [11] – and several TNBC cell lines [12, 13]. This potentiation occurs via activation of sphingosine kinase 1 (SphK1) and generation of sphingosine-1-phosphate (S1P) [12, 13], a lipid mediator of oncogenesis [14]. Direct binding of IGFBP-3 to EGFR, and their nuclear colocalization, in TNBC cells are enhanced in response to DNA-damaging chemotherapy [15]. ER-negative breast tumors also typically have higher expression of IGFBP-3 mRNA [16] and protein [17] than ER-positive tumors, and in women with breast cancer of the basal-like subtype, high IGFBP-3 is significantly associated with poorer recurrence-free survival [18]. Finally, SphK1 is also more highly expressed in ER-negative/TNBCs than in ER-positive breast cancer, and is significantly related to poorer disease-free survival [19].

Since IGFBP-3, which is abundant in the circulation as well as the cellular environment, is challenging to target, the strong associations among IGFBP-3, EGFR and SphK1 prompted us to evaluate co-targeting of the IGFBP-3 mediators EGFR and SphK1 in TNBC cell lines in vitro and in xenograft tumors. The SphK1 inhibitor, SKI-II, showed a powerful combined inhibitory effect with the EGFR tyrosine kinase inhibitor (EGFR-TKI) gefitinib (Iressa, AstraZeneca, Cambridge, UK), with significant inhibitory action under conditions where neither single agent was significantly inhibitory [13]. To translate this finding clinically it would be advantageous to use drugs in current clinical practice, with known toxicity and pharmacokinetics. FTY720 (Fingolimod, Novartis, Basel, Switzerland) is a structural analogue of sphingosine with S1P receptor 1 (S1P_1_) modulatory activity, that has clinical benefit in relapsing-remitting multiple sclerosis owing to its ability to inhibit T-cell migration from lymph nodes [20]. Among a range of other activities, FTY720 is also an inhibitor of SphK1 and has been proposed as a potential cancer therapy [21–24].

In this study, we demonstrate a powerful synergistic inhibition of TNBC cell growth by treatment with a combination of EGFR-TKI and FTY720, and show that, despite its potential immunomodulatory effect, FTY720 is effective in extending survival in both immune-deficient xenograft and immune-competent syngeneic mouse models.

## Methods

### Reagents, drugs and cell lines

Human TNBC cell lines, and the murine 4T1 mammary carcinoma cell line, were obtained from ATCC, Manassas, VA, USA and maintained in RPMI 1640 medium containing 5% FBS and 10 μg/mL bovine insulin under standard conditions. Identity of Hs578T cells, which were obtained from ATCC in 2001, was confirmed by short-tandem repeat profiling by CellBank Australia (Westmead, NSW, Australia) in December 2012. Cryopreserved stocks of other cell lines (purchased in 2010 from ATCC) were established within 1 month of receipt, and fresh cultures for use in experiments were established from these stocks every 2-3 months. All cell lines tested negative for mycoplasma contamination. Stable downregulation of IGFBP-3 was achieved by transfecting 2 × 10^6^ cells with SureSilencing shRNA or negative control plasmids according to the manufacturer’s instructions (Qiagen, Melbourne, VIC, Australia) and selection using hygromycin B. Cell lysates and media were collected post-selection to validate knockdown efficiency by qRT-PCR, radioimmunoassay and immunoblotting as previously described [13].

Gefitinib (Iressa; ZD1839, AstraZeneca) and FTY720 (Fingolimod; Gilenya, Novartis) were obtained from MedChem Express, Monmouth Junction, NJ, USA. Desmethyl erlotinib (OSI-420) was obtained from AdooQ BioScience, Irvine, CA, USA.

### Cell growth studies

Cell proliferation was studied using the IncuCyte live-cell imager (Essen BioScience, Ann Arbor, MI, USA) as previously described [13]. Cells (1 × 10^3^/well for 4T1, 2 × 10^3^/well for Hs578T, 4 × 10^3^/well for HCC70, HCC1806, MDA-MB-231 and MDA-MB-436, and 8 × 10^3^ cells/well for MDA-MB-468) were dispensed into 96-well plates in complete medium and incubated overnight before changing to fresh medium containing 5% FBS and inhibitors. Plates were transferred to the IncuCyte, and incubations continued over 72–136 h, depending on the cell line, with images collected every 3 h.

### Mouse models of TNBC

All animal procedures were approved by the institutional Animal Ethics Committee (Protocols RESP/14/280 and RESP/15/103). Female BALB/c nude mice, 6–7 weeks old, were obtained from Animal Resource Centre, Murdoch, Western Australia, and were acclimatized under local conditions before use as hosts for tumor xenografts at 8 weeks. Wildtype BALB/c mice were obtained from the institutional animal facility. For all tumors, cells were mixed with 50 μL Matrigel (BD Biosciences, Franklin Lakes, NJ, USA) and injected in 150 μL total volume into the 4th left mammary fat pad. Tumor volumes were measured three times weekly and mice were weighed weekly. All treatments were administered intraperitoneally (i.p.) three times weekly.

### MDA-MB-468 tumors

5 × 10^6^ MDA-MB-468 human mammary carcinoma cells were implanted in nude mice. When tumors reached 100 mm^3^ (length × width^2^/2), mice were randomized into groups of five mice for treatment. Protocol 1: FTY720 at 3 mg/kg (or vehicle) and gefitinib at 50 mg/kg (or vehicle). All mice were terminated on day 25 of treatment, the day when the largest tumor reached 500 mm^3^. Protocol 2: FTY720 at 5 mg/kg (or vehicle) and gefitinib at 25 or 50 mg/kg (or vehicle). Each mouse was terminated when its tumor reached 500 mm^3^, or at treatment day 40.

### HCC1806 and HCC70 tumors

5 × 10^6^ HCC1806 or HCC70 human mammary carcinoma cells were implanted in nude mice. When tumors reached 200 mm^3^ (100 mm^3^ for HCC70), mice were randomized into four groups of ten mice for treatment: FTY720 at 5 mg/kg and gefitinib at 25 mg/kg (50 mg/kg for HCC70). Each mouse was terminated when its tumor reached 1000 mm^3^ or at 12 weeks after injecting cells.

### 4T1 tumors

1 × 10^5^ 4T1 murine mammary carcinoma cells were implanted into BALB/c nude or BALB/c wild-type mice. When tumors reached 100 mm^3^, mice were randomized into four groups of ten mice for treatment: FTY720 at 5 mg/kg and gefitinib at 50 mg/kg. Each mouse was terminated when its tumor reached 1000 mm^3^.

### Analytical methods – cell culture

*IGFBP3, SPHK1* and *CD44* mRNA expression was measured, in duplicate, on duplicate RNA extracts by qRT-PCR as previously described [13], using the following Taqman probes (Applied Biosystems, Foster City, CA, USA): IGFBP-3: Hs00181211_m1; SPHK1: Hs00184211_m1; CD44: Hs01075864_m1; and HMBS (reference gene): Hs00609297_m1. IGFBP-3 concentrations in cell-conditioned media were measured by in-house radioimmunoassay [13]. Western blotting was performed as described previously [13] using antibodies from Cell Signaling Technology (Beverly, MA, USA): total EGFR (#2232, 1:1,500), pEGFR (Tyr1068) (#2234, 1:1,500), HER2/ErbB2 (#2242, 1:1,000), type 1 IGF receptor (IGF1R, #3027, 1:1,000), p53 (#9282, 1:1,000), p63 (#4892, 1:1,000), CD44 (#3570, 1:1,500), vimentin (#3390, 1:1,000), E-cadherin (#3195, 1:1,000). SphK1 antibody (ab16491, 1:1,000) was from Abcam (Walnut, CA, USA) and α-tubulin antibody (T9026, 1:10,000) from Sigma-Aldrich, St Louis, MO, USA. Rabbit antihuman IGFBP-3 antiserum R-100 was raised in-house. Secondary antibodies were from Pierce Biotechnology (Rockford, IL, USA).

### Analytical methods – tissue sections

Tumor samples were fixed in 10% phosphate-buffered formalin and embedded in paraffin. Four-micron sections were incubated with antibodies against Ki67 (ab66155, 1:600, Abcam, Melbourne, VIC, Australia), cleaved caspase-3 (Asp175) (#9661, 1:200, Cell Signaling), pEGFR (Tyr1068) (#2234, 1:300, Cell Signaling), SphK1 (#AP7237c, 1:400, Abgent, San Diego, CA, USA), CD44 (156-3C11, mouse mAb #3570, 1:200, Cell Signaling), IGFBP-3 (in-house antiserum R-100, 1:2000), or CD3 (ab16044, 1 μg/ml, Abcam) and isotyped-matched IgG antibodies. Immunodetection used the Dako EnVision + System-HRP labeled polymer detection kit (Dako, Carpinteria, CA, USA) with visualization using ImmPACT NovaRED Peroxidase (HRP) Substrate (# SK-4805, Vector Laboratories, Burlingame, CA, USA), and counterstaining by Mayer’s hematoxylin and Scott’s bluing solution. After mounting, sections were viewed by light microscope (Eclipse 80i, Nikon, Tokyo, Japan) and evaluated. For each antibody, all immunohistochemistry (IHC) was performed in a single assay to exclude between-run variability. Markers were evaluated by semi-quantitative scoring from the intact cell surface area from the five highest staining areas at × 10 magnification for each slide.

IHC staining of Ki67, cleaved caspase-3, IGFBP-3 and CD44 were scored as percentage of positive cells. Specific IGFBP-3 staining was mostly in the nucleus, and only nuclear staining was scored. For cleaved caspase-3 scoring, central necrotic parts of tumors were excluded. SphK1 and pEGFR were evaluated by both the percentage of target cells stained, and the staining intensity. The intensity was scored as 0 (no staining), 1 (weak), 2 (moderate), or 3 (strong). The final score, ranging from 0–300, was obtained by multiplying the scores for intensity by the percentage of positive cells.

### Data analysis and statistics

CompuSyn v1.0 software (ComboSyn Inc., Paramus, NJ, USA) was used to calculate the Chou-Talalay Combination Index (CI), where CI < 1indicates synergism and CI > 1 indicates antagonism [25]. All other statistical analyses were performed using SPSS v.22 for Mac (IBM Corp, Armonk, NY, USA). Effects of drug treatment on tumor IHC staining scores were calculated using 1-factor ANOVA followed by Tukey’s post hoc test. Pearson’s correlations among IHC staining scores are reported with two-tailed *P* values.

## Results

### Characterization of TNBC cell lines

**Fig. 1:**
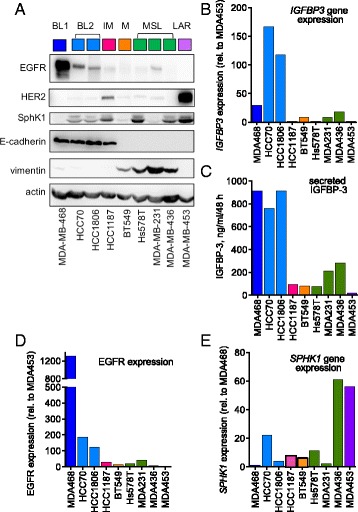
Characterization of TNBC subtype cell lines. **a** Lysates prepared from cells grown for 48 h in medium containing 10% FCS were evaluated for EGFR, HER2, SphK1, E-cadherin, and vimentin expression by western blot. Actin was measured as a loading control. The TNBC subtypes are basal-like 1 (BL1), basal-like 2 (BL2), immunomodulatory (IM), mesenchymal (M), mesenchymal stem-like (MSL), and luminal androgen receptor (LAR). Representative images from four similar experiments. **b** Relative IGFBP-3 mRNA levels averaged from duplicate cultures, normalized to MDA-MB-453 cells, analyzed by qPCR as described in Methods. **c** Secreted IGFBP-3 measured in duplicate by RIA specific for primate IGFBP-3 in 48-h conditioned medium (representative data from one of four similar experiments). **d** EGFR levels quantitated from the western blot in panel A, expressed relative to MDA-MB-453 cells. **e** Relative SphK1 mRNA levels averaged from duplicate cultures, normalized to MDA-MB-468 cells, analyzed by qPCR as described in Methods. *EGFR* epidermal growth factor receptor, *HER2* human epidermal growth factor receptor-2, *IGFBP-3* insulin-like growth factor binding protein-3, *SphK1* sphingosine kinase 1

Nine cell lines representing the six molecular subtypes of TNBC [5] were characterized for their expression of cellular markers. Basal-like cell lines (BL1: MDA-MB-468; BL2: HCC70, HCC1806) had much higher IGFBP-3 and EGFR expression than cells of the other subtypes (Fig. 1a-d). These cell lines, also classified as Basal A, strongly expressed the epithelial marker E-cadherin, had low vimentin expression, and low-to-moderate SphK1 protein and mRNA (Fig. 1a, e). The mesenchymal (BT549) and mesenchymal stem-like (MSL) (Hs578T, MDA-MB-231, MDA-MB-436) lines expressed high vimentin and undetectable E-cadherin, with much lower IGFBP-3 and EGFR levels than the BL1 and BL2 lines, and variable SphK1 expression. These lines are also classified as Basal B. HCC1187 cells (IM subtype) and MDA-MB-453 cells (luminal androgen receptor subtype) had relatively low IGFBP-3 and EGFR, and both showed substantial HER2 expression (Fig. 1a), contrary to their classification as triple-negative [5]. These two TNBC subtypes were not investigated further.

### A combination of FTY720 and EGFR-TKI acts synergistically to inhibit TNBC cell growth

**Fig. 2:**
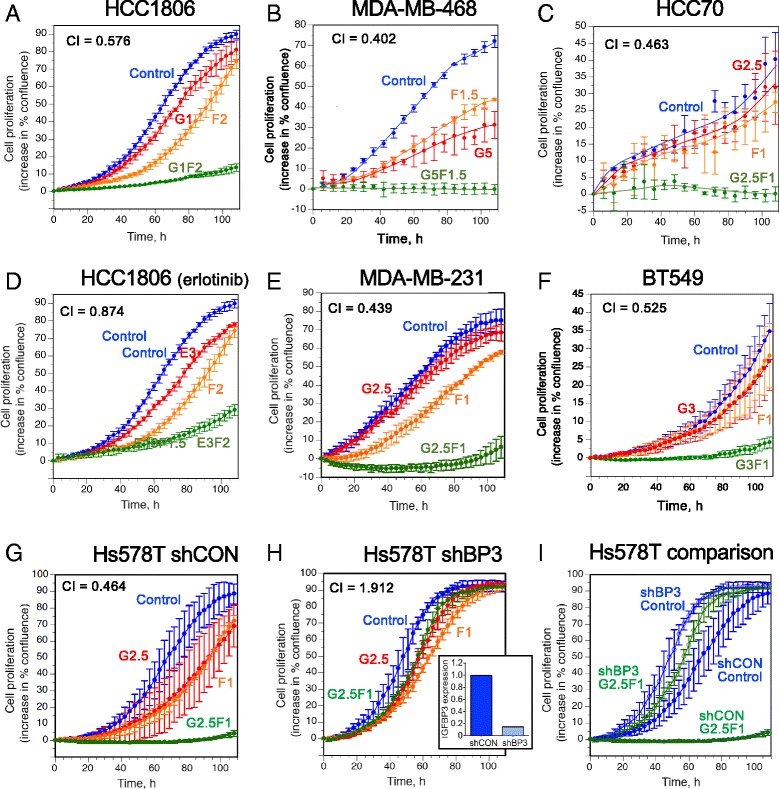
The effect of EGFR inhibitors and FTY720 on cell proliferation measured by IncuCyte real-time imaging. Data are expressed as the change in percent confluence, corrected for confluence at time zero, and are mean values ± SD at each time point, generally from triplicate wells for each treatment. Treatments are: control (*blue*), gefitinib (*red*, except in panel (**d**), erlotinib), FTY720 (*orange*), and both drugs combined (*green*). Drug concentrations (μM) for each cell line are designated as follows: G1 = gefitinib, 1 μM; E3 = erlotinib, 3 μM; F1 = FTY720, 1 μM; G1F1 = gefitinib, 1 μM + FTY720, 1 μM, etc. CI = combination index, a measure of drug synergy, calculated according to Ref. 25. Mammary carcinoma cell lines are: **a** HCC1806; **b** MDA-MB-468; **c** HCC70; **d** HCC1806, treated with erlotinib in place of gefitinib; **e** MDA-MB-231; **f** BT549; **g** Hs578T cells stably expressing empty shRNA vector (shCON); **h** Hs578T cells stably expressing IGFBP-3 shRNA (shBP3). Panel (**h**) *inset*: relative IGFBP-3 mRNA levels in Hs578T cells stably expressing shRNA vector or IGFBP-3 shRNA. **i** superimposed curves for control (no treatment) and G2.5 F1 (combination treatment) in shCON (*solid circles*) and shBP3 (*open circles*) Hs578T cell lines. *IGFBP-3* insulin-like growth factor binding protein-3

Figure 2a, b shows the effect of FTY720-gefitinib combinations on the proliferation of two human TNBC cell lines, HCC1806 (BL2) and MDA-MB-468 (BL1). At concentrations of gefitinib, optimized for each cell line, that had a modest effect on proliferation, FTY720 at a mildly cytostatic dose induced almost complete cytostasis when used in combination. Calculation of the Chou-Talalay combination index (CI) indicated strong synergism (CI < 1) between the two inhibitors in both cell lines. Similar synergistic inhibition was seen in the other BL2 cell line, HCC70 (Fig. 2c), the MSL line MDA-MB-231 (Fig. 2e), and the mesenchymal subtype cell line BT549 (Fig. 2f). Tested in the HCC1806 cell line, an alternative EGFR-TKI, erlotinib, showed lower synergy than gefitinib with FTY720 in inhibiting proliferation (Fig. 2d).

The contribution of IGFBP-3 in driving the synergistic proliferative pathways blocked by these inhibitors was demonstrated using cell lines in which IGFBP-3 was stably downregulated by IGFBP-3 shRNA. Figure 2g shows the synergistic effect of the FTY720-gefitinib combination in Hs578T cells (MSL subtype) stably transfected to express a control (non-silencing) shRNA (shCON). Similar to the other TNBC lines, doses of gefitinib and FTY720 that were modestly cytostatic individually, caused a strongly synergistic inhibition of cell proliferation when used in combination. In contrast, in Hs578T cells stably expressing shRNA against IGFBP-3 (shBP3) where IGFBP-3 expression was decreased by 75–85% measured as mRNA (not shown) or secreted IGFBP-3 (inset), the combined drugs showed no synergism (Fig. 2h), and in fact appeared antagonistic (CI > 1). In Fig. 2i, control and drug-combination curves are superimposed for the shCON and shBP3 cell lines, emphasizing that, while the shBP3 cells proliferated slightly faster than the shCON cells, they were almost completely insensitive to the drug combination. The combination effect was also decreased in HCC1806 clones with stable IGFBP-3 downregulation (see Additional file 1), although the high IGFBP-3 production by this cell line (Fig. 1b) made this more difficult to demonstrate. These observations are consistent with the premise that high IGFBP-3 expression in TNBC cell lines is a major driver of cell proliferation through the EGFR and S1P pathways [13], and suggests that since high tumor IGFBP-3 expression is required for the drugs to act synergistically, tissue IGFBP-3 might be evaluated as a biomarker for the efficacy of EGFR-TKI-FTY720 combination therapy.

**Fig. 3:**
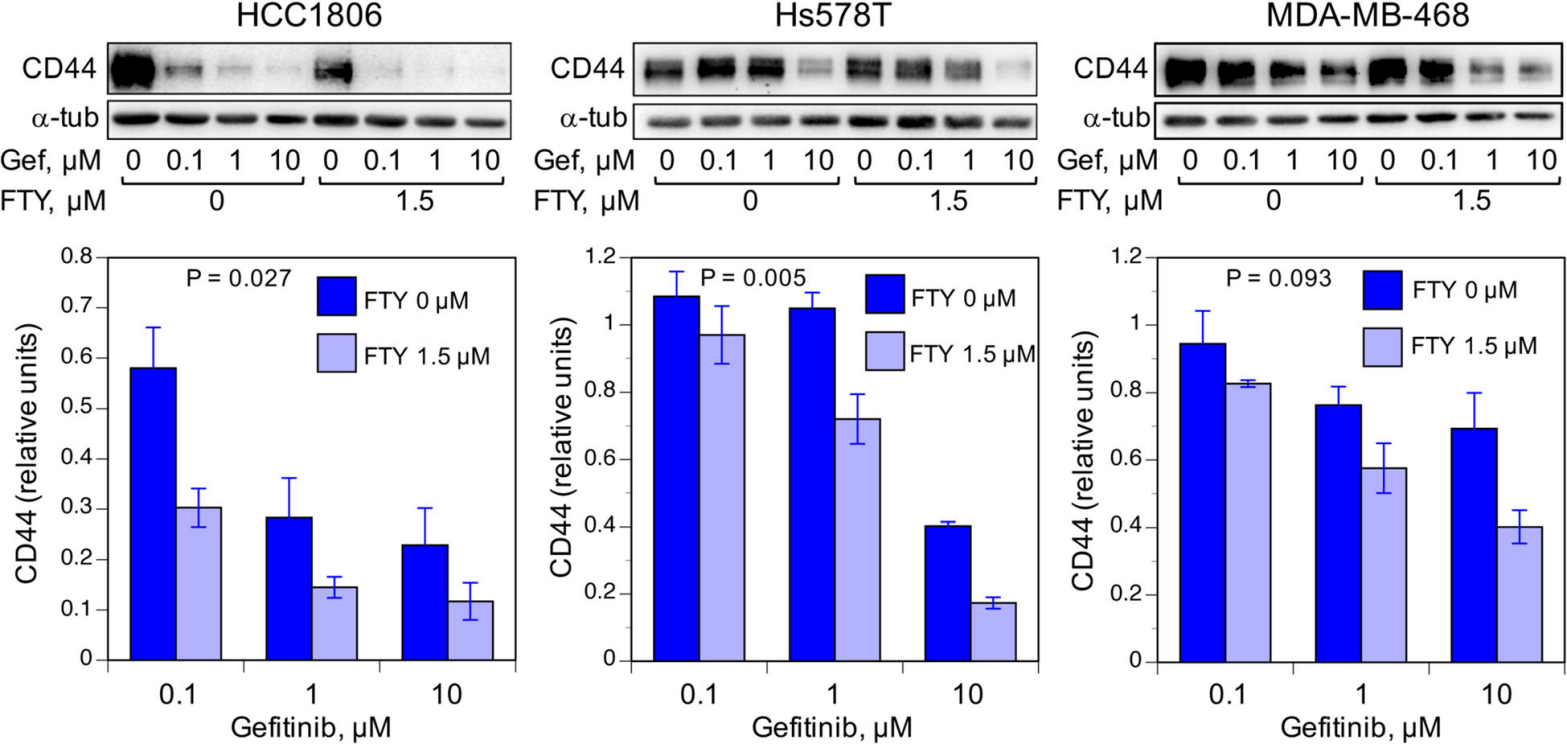
The effect of gefitinib-FTY720 treatment on CD44 expression in TNBC cell lines. HCC1806, Hs578T, and MDA-MB-468 TNBC cell lines at ~40% confluence in 12-well plates were exposed for 24 h to gefitinib (Gef: 0.1 to 10 μM), with or without the addition of 1.5 μM FTY720, as indicated. *Upper panels*: detection of standard isoform (~80 kDa) CD44 in cell lysates by western blotting. α-Tubulin (α-tub) is shown as a loading control. *Lower panels*: quantitation of the ~80-kDa band density was normalized to band density for cells receiving 0 gefitinib. Bars are mean value ± SEM for six (HCC1806), six (Hs578T) or three (MDA-MB-468) replicate samples. *P* values indicate significance of the effect of FTY720 addition, calculated over the three gefitinib doses by repeated measures ANOVA

In esophageal squamous cell carcinoma, like TNBC, IGFBP-3 is highly expressed and is associated with poor patient survival [26]. In esophageal squamous cell carcinoma cells in vitro, IGFBP-3 induces cells expressing high levels of CD44 [26], a transmembrane glycoprotein involved in tumor growth and progression [27]. Additional file 2 shows that, in four HCC1806 clones with varying IGFBP-3 mRNA levels, CD44 expression and IGFBP-3 expression were highly associated. Since both SphK1 [28] and EGFR [29] are reported to upregulate CD44, we proposed that they might act through CD44 to mediate the growth-regulatory effect of IGFBP-3 in TNBC. Figure 3 shows the effect of co-treatment of three TNBC cell lines with gefitinib and FTY720. All cell lines showed downregulation of ~80-kDa CD44 (standard isoform) by gefitinib at doses between 0.1 and 10 μM, with the inhibitory effect enhanced in the presence of 1.5 μM FTY720. The potentiating effect of FTY720 on CD44 downregulation was significant for HCC1806 and Hs578T cells, but not in MDA-MB-468 cells, for which fewer replicates were analyzed. Since high CD44 expression is a poor prognostic indicator in women with TNBC [30], our data suggest that a decline in CD44 expression caused by combination gefitinib-FTY720 treatment might contribute to improved patient survival.

### A combination of FTY720 and EGFR-TKI increases survival in mouse xenograft models of TNBC

**Fig. 4:**
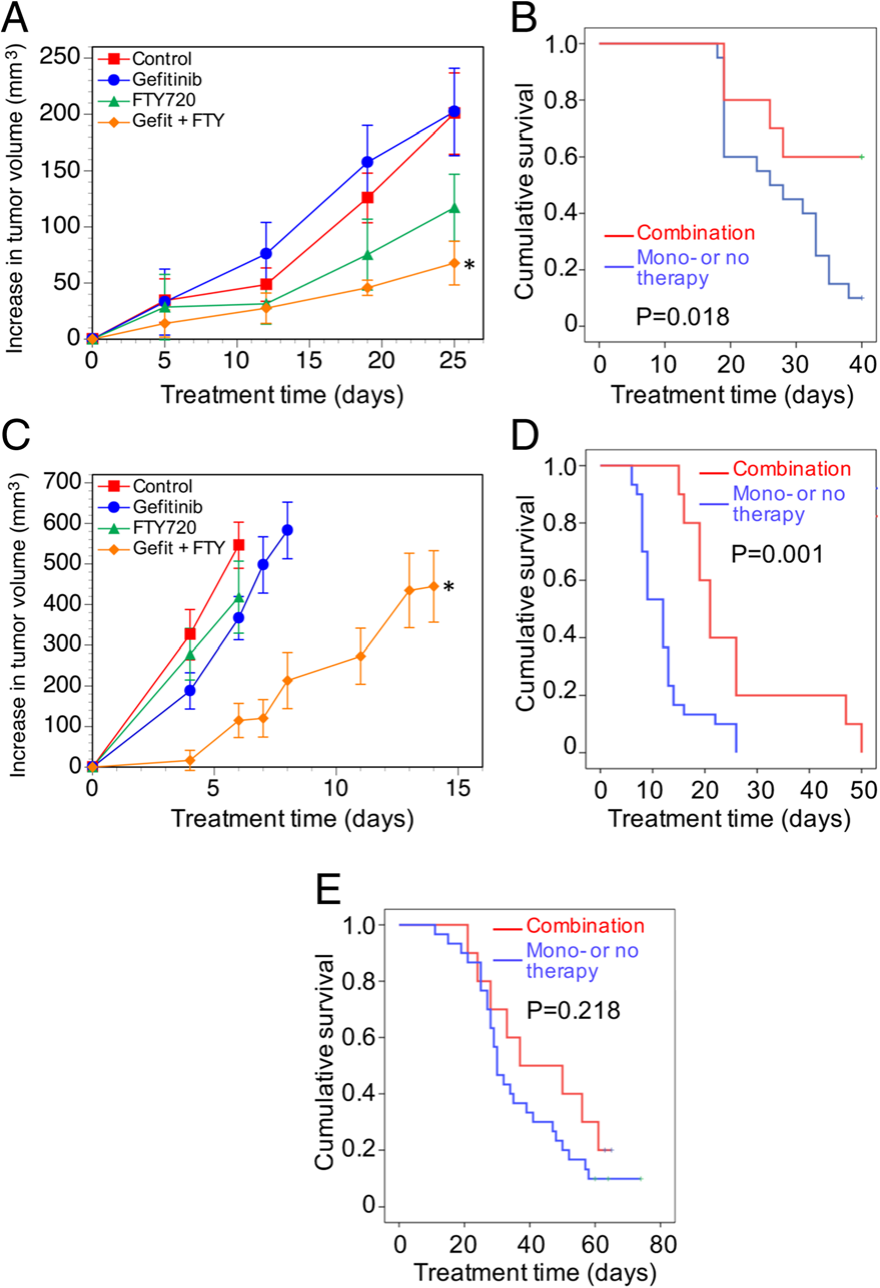
The effect of gefitinib-FTY720 combination therapy on TNBC xenograft tumor growth in nude mice. **a** Mean increases ± SEM (n = 5) in tumor volume from the commencement of treatment, 28 days after MDA-MB-468 cell implantation (tumor size ~100 mm^3^). All mice were sacrificed on day 25, the day the largest tumor reached 500 mm^3^ (Protocol 1). Gefitinib: 50 mg/kg; FTY720: 3 mg/kg. There is a significant effect of treatment overall (*P* = 0.044, repeated measures ANOVA). At sacrifice the increase in control-treated tumors was threefold greater than that of combination-treated tumors (^*^, *P* = 0.041, one-way ANOVA) but not significantly different from either monotherapy. **b** Kaplan-Meier analysis for survival effect of combination treatment, compared to control treatment and either monotherapy: log-rank *P* = 0.018. Treatment (gefitinib, 25 or 50 mg/kg; FTY720, 5 mg/kg) in groups of five mice commenced 28 days after MDA-MB-468 cell implantation (tumor size ~100 mm^3^) and each mouse was sacrificed when its tumor reached 500 mm^3^ (Protocol 2). Data are combined for gefitinib at 25 or 50 mg/kg. **c** For HCC1806 cell tumors, treatment (gefitinib, 25 mg/kg; FTY720, 5 mg/kg) commenced 7 days after cell implantation, (tumor size ~200 mm^3^). Each mouse was sacrificed when its tumor reached 1000 mm^3^; mean increases ± SEM (n = 10) in tumor volume are shown for time points until the first mouse in each group was culled. Overall effect of treatment: *P* = 0.002, repeated measures ANOVA. The increase in combination-treated tumors is significantly less than controls (^*^ *P* = 0.010), gefitinib-treated (*P* = 0.006), and FTY720-treated (*P* = 0.026) tumors (post hoc Tukey’s test). **d** Kaplan-Meier analysis from the experiment described in Fig. 2c. Survival effect of combination treatment compared to control-treated mice and those on either monotherapy: log-rank *P* = 0.001. **e** For HCC70 cell tumors, treatment (gefitinib, 25 mg/kg; FTY720, 5 mg/kg) in groups of ten mice commenced when tumor size was ~100 mm^3^ (about 14 days after implantation). Kaplan-Meier analysis for survival effect of combination treatment, compared to control mice and those on either monotherapy: log-rank *P* = 0.218

Three basal-like TNBC cell lines growing as orthotopic xenograft tumors in nude mice were tested for their responsiveness to gefitinib-FTY720 combination therapy. The protocols differed slightly for each cell line. Initially tumors grown from MDA-MB-468 cells (BL1) were treated with gefitinib at 50 mg/kg and FTY720 at 3 mg/kg when tumors reached approximately 100 mm^3^, and treatment continued until day 25 when the largest tumor reached a volume of 500 mm^3^ (Protocol 1). As shown in Fig. 4a, a significant effect of treatment on tumor growth was observed over the full treatment period (*P* = 0.044, repeated measures ANOVA). This was more highly significant over the final two treatment days (*P* = 0.009), when post hoc testing showed the increase in tumor size in mice receiving combination therapy was significantly less than that in control mice (*P* = 0.032), whereas there was no difference between control mice and those receiving either monotherapy. The drug combination was also tested on MDA-MB-468 tumors in a survival protocol (Protocol 2, Methods). Gefitinib was trialed at 25 and 50 mg/kg but, as there was no difference in mouse survival time, the data were combined. Figure 4b shows a Kaplan-Meier plot for mouse survival (censored at day 40 after treatment commenced) or death (defined as tumor reaching 500 mm^3^). Comparing the combination treatment (for both gefitinib doses) to no treatment or either monotherapy, gefitinib-FTY720 significantly enhanced survival time (log-rank *P* = 0.018).

For tumors from HCC1806 cells (BL2), treatment commenced when they reached approximately 200 mm^3^, and continued until each tumor reached 1000 mm^3^, which was used as the survival endpoint for Kaplan-Meier analysis. Figure 4c shows the mean increase in tumor volume for each treatment group, calculated until the first mouse in each group was culled (i.e., ten mice per data point). A highly significant effect of treatment was seen (*P* = 0.002, repeated measures ANOVA), and by post hoc testing (calculated until treatment day 8 owing to the decreasing numbers of animals), the increase in tumor volume in animals treated with the drug combination was significantly less than that for no treatment or either monotherapy. As seen in the Kaplan-Meier analysis (Fig. 4d), mice receiving the combination treatment had significantly extended survival compared to mice receiving no therapy or either monotherapy, from a mean of 12.3 ± 1.0 days to 26.0 ± 3.9 days (log-rank *P* = 0.001). In contrast, in mice bearing the more slow-growing HCC70 (BL2) cell tumors, the approximately 20% survival benefit from the combination treatment under the conditions tested (mean = 59.5 ± 5.9 days), compared to control mice and those on either monotherapy (50.5 ± 3.0 days) was not significant (log-rank *P* = 0.218) (Fig. 4e).

### The combination therapy is more effective in immune-competent than immune-deficient mice

**Fig. 5:**
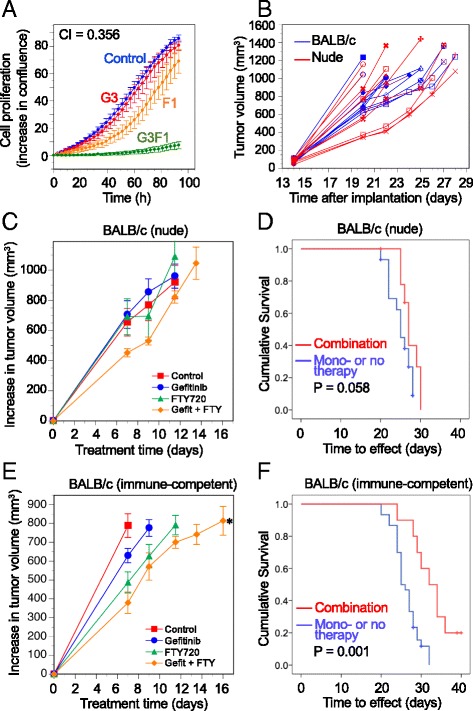
The effect of gefitinib-FTY720 therapy on syngeneic 4T1 mammary tumor growth in immune-deficient and -competent mice. **a** Gefitinib and FTY720 synergistically inhibit cell proliferation of 4T1 murine mammary carcinoma cells, measured by IncuCyte real-time imaging. Data are expressed as the increase in percent confluence, corrected for confluence at time zero, and are mean values ± SD at each time point for triplicate wells for each treatment. Treatments are: control (*blue*), gefitinib 3 μM (*red*), FTY720 1 μM (*orange*), and both drugs combined (*green*). CI = combination index. **b** Growth of 4T1 murine mammary tumors in untreated nude (immune-deficient) and wild-type (immune-competent) BALB/c mice. In both strains tumors took a mean of 24 ± 3 days after implantation to reach at least 1000 mm^3^. **c** In nude mice, single or combination therapy had no significant effect on tumor growth rate. *Red squares*: control; *blue circles*: gefitinib, 50 mg/kg; *green triangles*: FTY720, 5 mg/kg; *orange diamonds*: combination. **d** Kaplan-Meier survival analysis showed that gefitinib-FTY720 combination therapy did not significantly extend survival compared to mono- or no therapy (log-rank *P* = 0.058). **e** In wild-type mice, combination therapy, but neither monotherapy, significantly decreased tumor growth rate compared to no therapy (*P* = 0.041, Tukey’s post hoc test after repeated measures ANOVA). **f** Combination therapy significantly extended survival (log-rank *P* = 0.001) compared to mice receiving mono- or no therapy

The retention of lymphocytes in lymphoid tissue and their entry into the circulation is regulated in part by S1P [31]. In patients with relapsing multiple sclerosis, FTY720 is used for the ability of its phosphorylated product to downregulate the S1P_1_ receptor [20], and this results in lymphopenia in about 10% of multiple sclerosis patients [32]. In cancer patients, inhibition of lymphocyte mobilization might adversely affect therapeutic response [33]. To examine the importance of the immune system in the response to EGFR-TKI-FTY720 combination therapy, we compared the effect of the drugs in wild-type (immune-competent) BALB/c mice and nude (immune-deficient) BALB/c mice, using the syngeneic murine TNBC cell line 4T1. As shown in Fig. 5a, this cell line showed the same synergistic inhibition by gefitinib and FTY720 in vitro as the human TNBC cell lines shown in Fig. 2. The growth rate of untreated 4T1 tumors did not differ between mouse strains, taking a mean of 24 ± 3 days after implantation to reach 1000 mm^3^ in both strains (Fig. 5b). In BALB/c nude mice, the effect of treatment on the increase in tumor size (Fig. 5c) was not significant. Similarly, as shown in Fig. 5d, the survival advantage attributable to combination therapy compared to control or either monotherapy in the BALB/c nude mice (27.5 ± 0.7 vs. 25.0 ± 0.5 days) was not significant (log-rank *P* = 0.058). In contrast, in BALB/c mice with normal immune function, there was a significant effect of treatment on the increase in tumor size (*P* = 0.028, repeated measures ANOVA), and by post hoc testing (calculated until treatment day 14 owing to the decreasing numbers of animals), the increase in tumor size in mice receiving combination therapy was significantly less than that in control mice (*P* = 0.041), whereas there was no effect for either monotherapy (Fig. 5e). By Kaplan-Meir analysis (Fig. 5f), the survival advantage with the combination therapy (32.7 ± 1.6 days) was highly significant compared to no treatment or either monotherapy (26.2 ± 0.6 days, log-rank *P* = 0.001). This indicates that at a therapeutically active concentration of FTY720 (when combined with gefitinib), potential inhibition of T cell mobilization does not negate the survival benefit of the drug combination.

To investigate this further we measured CD3 levels, indicating T cell infiltration, in a subset of tumors from wild-type BALB/c mice. Treatment with FTY720 substantially depleted tumor T cells as indicated by decreased CD3, whether measured by immunoblot on tumor lysates (Additional file 3, panel A, B), or IHC on tumor sections (Additional file 3, panel C). Unexpectedly, in tumors from mice treated with the gefitinib-FTY720 combination, this depletion was greatly blunted, with CD3 levels not lower than those in control animals, perhaps contributing to the effectiveness of the combination in wild-type mice. No CD3 was detectable by IHC in sections from tumors grown in nude mice (not shown).

### Tumor immunohistochemistry

**Fig. 6:**
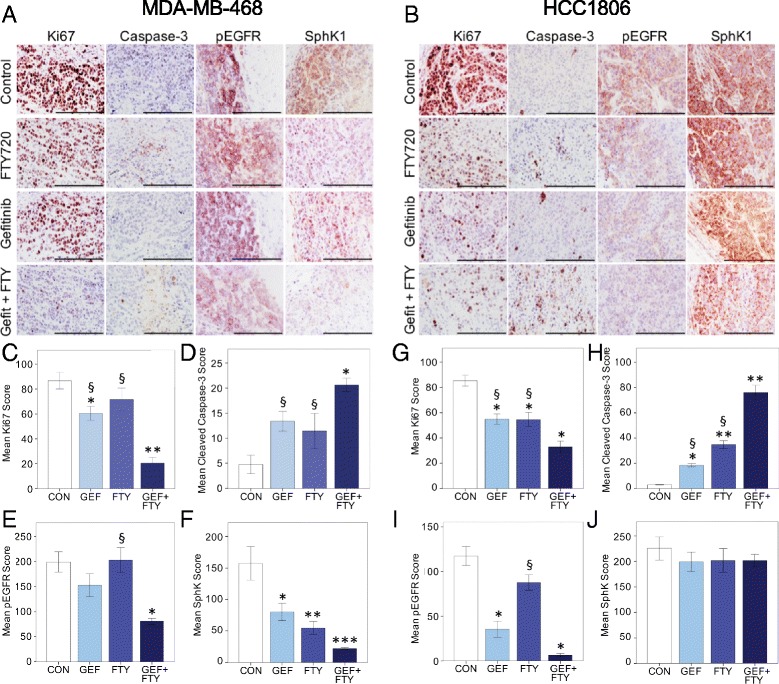
Immunohistochemical analysis of TNBC tumors from control, gefitinib-treated (GEF), FTY720-treated (FTY), and combination-treated mice. Tumors were stained for Ki67, cleaved caspase-3, pEGFR, and SphK1 as indicated. Scoring for each antigen was as described under Methods. **a** Representative staining of sections from MDA-MB-468 tumors. **b** Representative staining of sections from HCC1806 tumors. All summary data are mean values ± SEM, analyzed by one-way ANOVA followed by post hoc Tukey’s test. All ANOVA comparisons were highly significant for treatment except for SphK1 staining in HCC1806 tumors. Panels **c**-**f** MDA-MB-468 tumors. **c** Ki67 staining (n = 4–9 per group), ^*^ *P* < 0.05, ^**^ *P* < 0.001 compared to control; ^§^ *P* < 0.001 compared to combination. **d** Cleaved caspase-3 staining (n = 4–10 per group), ^*^ *P* < 0.001 compared to control; ^§^ *P* = 0.05 compared to combination. **e** pEGFR staining (n = 3–5 per group), ^*^ *P* < 0.02 compared to control; ^§^ *P* < 0.02 compared to combination. **f** SphK1 staining (n = 3–5 per group): ^*^ *P* < 0.025, ^**^ *P* = 0.005, ^***^ *P* = 0.001 compared to control. Panels **g**-**j** HCC1806 tumors. **g** Ki67 staining (n = 10 per group), ^*^ *P* < 0.001 compared to control; ^§^ *P* = 0.01 compared to combination. **h** Cleaved caspase-3 staining (n = 8–10 per group), ^*^ *P* = 0.005, ^**^ *P* < 0.001 compared to control; ^§^P < 0.001 compared to combination. **i** pEGFR staining (n = 9–10 per group), ^*^ *P* < 0.001 compared to control; ^§^ *P* < 0.001 compared to combination. **j** SphK1 staining (n = 6 per group). Bar = 200 μm. *pEGFR* phosphorylated epidermal growth factor receptor, *SphK1* sphingosine kinase 1

To evaluate the effect of the gefitinib and FTY720 on tumor tissue endpoints, IHC was performed on tumors at the end of the in vivo experiments. Staining was undertaken for Ki67 and cleaved caspase-3 to indicate tissue proliferation and apoptosis, and pEGFR and SphK1 as targets of the two inhibitors. Figure 6a and b show representative IHC images in tumors grown from MDA-MB-468 and HCC1806 cells, respectively. In tumors of both cell types, combination treatment with gefitinib and FTY720 significantly decreased cell proliferation (Ki67 staining) below levels in either control animals (*P* < 0.001) or animals treated with either single agent (Fig. 6c, g). The drug combination was also strongly pro-apoptotic, increasing cleaved caspase-3 significantly above levels seen in controls (*P* < 0.001) or after either monotherapy (Fig. 6d, h). Cleaved caspase-3 staining showed a significant inverse association with Ki67 staining in both HCC1806 (r = -0.602, *P* < 0.001) and MDA-MB-468 (r = -0.705, *P* < 0.001) tumors (Additional file 4, panel A, B).

Gefitinib treatment alone significantly decreased pEGFR staining in HCC1806 tumors (Fig. 6i) but not in MDA-MB-468 tumors (Fig. 6e). FTY720 alone had no effect on pEGFR in either tumor type, but the drug combination strongly suppressed pEGFR in both MDA-MB-468 and HCC1806 tumors. Higher pEGFR staining was significantly associated with higher proliferation (Ki67) in both tumor types (Additional file 4, panel C, D). Both gefitinib and FTY720 alone significantly downregulated SphK1 levels in MDA-MB-468 tumors, with the combination causing further suppression (Fig. 6f). In contrast, SphK1 staining in HCC1806 tumors was unaffected by either single drug, or the combination (Fig. 6j), suggesting that SphK1 downregulation is not part of the cytostatic mechanism in these tumors. Despite this, there was a significant association between proliferative activity (Ki67 staining) and SphK1 expression in both tumor types (Additional file 4, panel E, F).

Since SphK1 protein expression, measured by western blotting, has been found to correspond to EGFR phosphorylation in esophageal cancer cell lines [34], we also examined the relationship between SphK1 and pEGFR IHC staining. A significant association was seen in MDA-MB-468 tumors (r = 0.554, *P* = 0.026), but not in HCC1806 tumors (r = 0.058, NS) (data not shown). CD44 was also measured by IHC in both MDA-MB-468 and HCC1806 tumors, but no differences among the four treatment groups were seen for either tumor type (data not shown).

The nuclear abundance of IGFBP-3, which we propose is an “upstream” activator of the targeted EGFR and SphK1 pathways, was also examined in xenograft tumors. Additional file 5A shows the typical marked reduction in nuclear IGFBP-3 in response to each inhibitor, with a greater effect of the combined drugs. Additional file 5B shows the quantitation of nuclear IGFBP-3 staining for groups of six HCC1806 tumors from each treatment group, with a highly significant loss of nuclear IGFBP-3 in the combination-treated tumors. A similar trend in nuclear IGFBP-3 staining was seen in MDA-MB-468 tumors (not shown).

## Discussion

Combinations of receptor tyrosine kinase inhibitors have previously been tested in breast cancer cell lines, including TNBC cells [35]. In the present study, we have evaluated the novel combination of tyrosine kinase (EGFR) inhibition with lipid kinase (SphK1) inhibition for its ability to block IGFBP-3-dependent proliferative signaling in TNBC, based on the observations that: (i) IGFBP-3 is abundant in many TNBC tumors, and prognostic for poor patient survival, (ii) endogenous IGFBP-3 potentiates EGFR signaling and EGFR-dependent cell proliferation, (iii) IGFBP-3 activates the oncogenic lipid kinase SphK1, and (iv) the effects of IGFBP-3 on EGFR-dependent proliferation are blocked by sphingosine kinase inhibition or downregulation. IGFBP-3 is abundant in the circulation where it is the predominant transport protein for the growth factors, IGF-1 and IGF-2 [36]. Circulating IGFBP-3 is mainly found in ternary complexes with the acid-labile subunit (ALS), with limited egress from the vasculature. Thus tissue effects of IGFBP-3 may largely be attributable to local (tissue and tumor) production. In breast cancer tissue, IGFBP-3 is predominantly associated with ER-negative tumors, with much lower expression in ER-positive breast cancer [16]. Since modulating the tissue (autocrine or paracrine) effects of IGFBP-3 by blocking the abundant circulating protein would be therapeutically challenging, we co-targeted two kinases that mediate at least some of its tissue effects in TNBC, EGFR and SphK1.

Whereas EGFR kinase inhibitors, such as gefitinib and erlotinib, are in routine clinical use, the development of SphK1 inhibitors is less advanced [37]. FTY720, used clinically for immunomodulation in relapsing-remitting multiple sclerosis, also shows efficacy in preclinical cancer models [21, 24]. It has multiple molecular targets, of which the downregulation of S1P_1_ receptors, leading to inhibition of T-lymphocyte mobilization from lymphoid tissue, is perhaps the best characterized [20]. FTY720 also has well-characterized SphK1 inhibitory activity, for which it was selected in this study. However, since we have shown that S1P_1_ inhibition or downregulation prevented the potentiation of EGFR signaling by IGFBP-3 in the triple-negative, nonmalignant mammary cell line MCF-10A [12], S1P_1_ downregulation as well as SphK1 inhibition might contribute to FTY720 efficacy in our studies.

Gefitinib-FTY720 combinations were profoundly growth-inhibitory to a variety of TNBC cell lines in vitro, under conditions where neither drug alone was very inhibitory. At optimized drug combinations, strongly synergistic effects were observed. Of the representative TNBC cell lines tested, this strong combination effect was seen in every case, with no distinction among the four TNBC subclasses tested: BL1, BL2, mesenchymal or MSL, while the murine TNBC cell line 4 T1 showed a similar synergistic response. Evaluated in a single cell line (HCC1806), the EGFR kinase inhibitor erlotinib showed somewhat less synergy than gefitinib, with slightly higher doses needed for the same inhibitory effect. Although gefitinib is typically used clinically at a higher dose than erlotinib, owing to different pharmacokinetics [38], a higher IC_50_ value (lower efficacy) in vitro for erlotinib compared to gefitinib has been reported in several TNBC cell lines [39].

Both drug targets, EGFR and SphK1, are activated by a wide range of cell effectors, and it is clear that neither EGFR inhibitors nor SphK1 inhibitors uniquely target IGFBP-3 signaling. Yet the central role of IGFBP-3 in activating these pathways in TNBC is emphasized by the observation that stable downregulation of IGFBP-3 in Hs578T cells abolished, and in HCC1806 cells diminished, the synergistic interaction between the two drugs. This reinforces that tumor IGFBP-3 expression is involved in driving EGFR-SphK1-dependent proliferation in TNBC cells, and should be evaluated as a possible biomarker of efficacy of the inhibitory drug combination. Interestingly, nuclear IGFBP-3 abundance showed highly significant downregulation by the inhibitor combination. IGFBP-3 has well-documented roles in the nucleus [40], and nuclear IGFBP-3 has been reported as a strongly negative prognostic indicator in prostate cancer [41]. Although the precise mechanism of nuclear IGFBP-3 downregulation in response to gefitinib-FTY720 co-treatment is unknown, this effect would be expected to contribute to the antitumor actions of the combination treatment.

The transmembrane cell adhesion molecule, CD44, is known to promote tumorigenic signaling in breast cancer. It exists in a number of splice variant isoforms, of which the ~80-kDa form is known as standard CD44, lacking the extracellular-domain inserts found in larger variants [27]. CD44 is recognized as a cancer stem cell marker and is enriched in basal-like breast cancers [42]. Its high expression is associated with poor disease-free survival in women with TNBC [30]. Because IGFBP-3 has been identified as a positive regulator of CD44-high cells [26], and the IGFBP-3 downstream mediators, EGFR and SphK1, are also known to regulate CD44 [28, 29], we examined CD44 abundance in TNBC cells treated with the drug combination. In three TNBC cell lines, CD44 downregulation by gefitinib was potentiated by the addition of FTY720, suggesting CD44 as a plausible intermediate in a growth-stimulatory signaling cascade that is inhibited by dual EGFR-SphK1 blockade. In vivo, however, no changes in total tumor CD44 were seen among treatment groups by IHC. Since only the ~80-kDa CD44 isoform was measured by western blot in cell lysates, but most or all of the multiple variant forms would be detected by IHC, it is possible that changes in regulated isoforms might be masked by other abundant isoforms comprising total tissue CD44. Despite the lack of posttreatment downregulation seen in vivo, it will be important to determine in prospective studies whether high CD44 expression, already identified as a prognostic indicator in TNBC, might have value in predicting responsiveness to the combination therapy.

When tested in xenograft models in nude mice, the gefitinib-FTY720 combination significantly inhibited the growth of MDA-MB-468 and HCC1806 tumors, and extended the survival of mice bearing these tumors, compared to vehicle treatment or either monotherapy. In contrast, the survival of mice bearing HCC70 tumors was not significantly extended by the combination treatment. Since HCC70 and HCC1806 cells are comparable in IGFBP-3, EGFR and SphK1 expression, yet the treatment was most effective in HCC1806 tumors and least effective in HCC70 tumors (despite using the higher gefitinib dose of 50 mg/kg), additional factors may regulate treatment efficacy. Our cell proliferation studies indicated that, of the three cell lines, gefitinib sensitivity was highest in HCC1806 and lowest in HCC70 (data not shown), reflecting the effectiveness of the treatments in vivo, and suggesting that an even higher gefitinib dose may have been more effective in treating HCC70 tumors. Since initial trials in MDA-MB-468 tumors showed no difference between gefitinib at 25 or 50 mg/kg, three times weekly, subsequent xenograft tumors, HCC1806 and HCC70, were treated with gefitinib at 25 mg/kg. Other mouse studies have used doses up to 150 mg/kg/day orally for 5 days per week [43, 44]; thus our dose of 25 mg/kg, chosen to demonstrate synergy with FTY720, was relatively low.

FTY720 was used at 3 or 5 mg/kg, three times weekly, in our combinations. This is also conservative for comparable animal studies, since other xenograft tumor studies have used doses up to 10 mg/kg every 2 days [45, 46]. FTY720 has not been trialed in human cancer patients, but compared to the standard immunomodulatory dose of 0.5 mg/day for patients with multiple sclerosis [32] – less than 0.01 mg/kg – doses used in cancer xenograft studies are extremely high. It is unclear why mice show such marked insensitivity to this drug compared to humans. Nevertheless, in the context of a possible clinical trial of the gefitinib-FTY720 combination in breast cancer patients, we investigated whether a FTY720 dose with antitumor efficacy in mice (in combination with gefitinib) would compromise the immune system. FTY720 inhibits T cell mobilization from lymphoid tissues [20], which in some patients can result in lymphopenia [32, 47]. If this depletes tumor-resident lymphocytes it could potentially be deleterious to breast cancer patients undergoing therapy, since a high tumor-associated lymphocyte count is a positive predictor of treatment response [48].

Since this could not be examined in nude mice which lack mature T lymphocytes, we grew the murine mammary carcinoma cell line, 4T1, in the syngeneic mouse strain BALB/c, allowing us to compare the gefitinib-FTY720 combination in immune-deficient (nude) versus immune-competent (wild-type) mice. The hypothesis was that, since tumor-associated T cells are associated with improved treatment response, we would see greater drug efficacy in the immune-competent mice only if their immune system was not compromised by FTY720. Our observation of a highly significant extension of survival by the gefitinib-FTY720 combination in wild-type mice, compared to a nonsignificant effect in nude mice, suggests that FTY720 at a therapeutically active dose (in combination with gefitinib) did not prevent the enhancing effect of tumor lymphocytes on drug efficacy. A previous study suggested that, although FTY720 impairs T cell trafficking from lymphoid tissues, it does not appear to compromise the beneficial activity of intratumoral T cells [49]. However, in a limited sample set, we saw a substantial decrease in tumor CD3 upon FTY720 treatment. Gefitinib therapy in patients has also been associated with lymphopenia [50]. Surprisingly, in tumors treated with the gefitinib-FTY720 combination, the decline in tumor CD3 was greatly alleviated. While the mechanism of this combination effect remains to be determined, this suggests that FTY720, in combination with gefitinib, could be explored at its approved immunomodulatory dose of 0.5 mg/day, for efficacy in women with TNBC.

Immunohistochemical analysis of tumor tissues indicated that in mice bearing either MDA-MB-468 or HCC1806 xenograft tumors, the gefitinib-FTY720 combination was significantly more antiproliferative (decreased Ki67) and more pro-apoptotic (increased cleaved caspase-3) than either drug alone. This supports the cell culture data as well as the in vivo tumor growth and mouse survival data. Gefitinib shows strong selectivity for EGFR kinase activity, although there is also weak cross-reactivity with HER2 [51]. In these triple-negative breast tumors the effect is likely to be entirely attributable to EGFR inhibition, and was reflected by significant inhibition of pEGFR by gefitinib treatment in HCC1806 tumor tissue, although the inhibition by gefitinib alone was not significant in MDA-MB-468 tumors. FTY720 treatment alone did not decrease pEGFR in either tumor type, but strongly enhanced the inhibitory effect of gefitinib. This potentiating effect of FTY720 on EGFR kinase inhibition has not previously been reported, although a combination effect of FTY720 (used as an S1P receptor antagonist) and the EGFR monoclonal cetuximab has been reported in colorectal cancer cells and xenograft tumors [52].

## Conclusions

In summary, our data show strongly synergistic growth inhibition by gefitinib and FTY720 in human cell lines representing the major TNBC subtypes, and prolonged survival in mice bearing TNBC xenograft tumors after treatment with the drug combination. In vitro studies suggest that IGFBP-3, which potentiates EGFR signaling via activation of SphK1, is a driver of proliferation in these tumors and its measurement in tumor tissue should be evaluated as a potential biomarker of drug combination efficacy. Immune-competent mice show greater responsiveness to the combination therapy than immune-deficient mice, indicating that FTY720 at a therapeutically active dose (in combination) does not block the tumor-suppressive effect of the immune system. We therefore propose that this drug combination could be evaluated for efficacy in women with basal-like TNBC tumors, potentially offering a treatment with lower toxicity than standard chemotherapy.

## Additional files

Additional file 1: Figure S1. The effect of gefitinib-FTY720 combination on proliferation of HCC1806 cells with stable IGFBP-3 knockdown. (A) Decreased IGFBP-3 production by two HCC1806 clones stably expressing shIGFBP-3 (shBP3) compared to two control clones expressing vector alone. (B) Cell proliferation data for combined shBP3 clones (*red*) compared to combined control clones (*blue*). Although shBP3 cells proliferate identically to control cells in the absence of treatment (*solid symbols*), treatment with the gefitinib-FTY720 combination (*open symbols*) is much more inhibitory to control cells than to cells with decreased IGFBP-3 expression. (C, D) Individual proliferation data for two HCC1806 control clones treated with gefitinib (2 μM), FTY720 (0.5 μM), or the combination. (E, F) Similar data for two HCC1806 shBP3 clones. Data are corrected for confluence at time zero, and are mean values ± SD at each time point for triplicate wells for each treatment. (PDF 420 kb)

Additional file 2: Figure S2. Relationship between IGFBP-3 mRNA and CD44 mRNA levels in HCC1806 clones with varying IGFBP-3 expression. Total RNA was extracted from duplicate cultures, reverse-transcribed and analyzed by qRT-PCR in duplicate as previously described [13]. Data points for each mRNA species were expressed relative to a single low-IGFBP-3 clone (B10) and were fitted using a four-parameter sigmoid logistic curve. (PDF 101 kb)

Additional file 3: Figure S3. CD3 levels in 4T1 tumors grown in wild-type BALB/c mice. Samples were from the experiment shown in Fig. 5d. (A) Western blot of three tissue lysates from each of the four treatment groups. α-Tubulin is shown as a loading control. (B) Quantitation of CD3 blots shown in Panel A, corrected for α-tubulin. Data are expressed as mean density values ± SEM. C. Immunohistochemistry of CD3 in three tumors in wild-type BALB/c mice from each of the four treatment groups, and one tumor in BALB/c nude mice from each treatment group as a negative control. (PDF 5088 kb)

Additional file 4: Figure S4. Correlations among immunohistochemical scores for cleaved caspase-3, Ki67, pEGFR and SphK1, in HCC1806 and MDA-MB-468 xenograft tumors. Data from the experiments are shown in Fig. 6. Correlations were assessed using Pearson’s correlation coefficient with two-tailed *P* values. (A, C, E) HCC1806 tumors. (A) cleaved caspase-3 vs. Ki67: n = 38, r = -0.602, *P* < 0.001. (C) Ki67 vs. pEGFR: n = 38, r = 0.576, *P* < 0.001. E: Ki67 vs. SphK1: n = 24, r = 0.462, *P* = 0.023. B, D, F: MDA-MB-468 tumors. (B) cleaved caspase-3 vs. Ki67: n = 25, r = -0.705, *P* < 0.001. D: Ki67 vs. pEGFR: n = 16, r = 0.726, *P* = 0.001. F: Ki67 vs. SphK1: n = 16, r = 0.614, *P* = 0.011. (PDF 308 kb)

Additional file 5: Figure S5. Immunohistochemical analysis of nuclear IGFBP-3 in HCC1806 tumors from control, gefitinib-treated (GEF), FTY720-treated (FTY), and combination-treated mice. (A) Tumors were stained for nuclear IGFBP-3, scored as described under Methods. Representative images from each treatment group (bar = 200 μm). (B) Summary data are mean values ± SEM (n = 6 per group), analyzed by one-way ANOVA followed by post hoc Tukey’s test. ^*^ *P* = 0.005, ^**^ *P* < 0.001 compared to control; ^§^ *P* < 0.025 compared to combination. (PDF 691 kb)

## Abbreviations

BL1: Basal-like 1
CI: Combination index
EGF: Epidermal growth factor
EGFR: EGF receptor
ER: Estrogen receptor
HER2: Human EGF receptor-2
IGFBP-3: Insulin-like growth factor binding protein-3
IHC: Immunohistochemistry
IM: Immunomodulatory
MSL: Mesenchymal stem-like
S1P: Sphingosine-1-phosphate
SphK1: Sphingosine kinase 1
TKI: Tyrosine kinase inhibitor
TNBC: Triple-negative breast cancer
