# Supplementary material for: Inhibition of basal-like breast cancer growth by FTY720 in combination with epidermal growth factor receptor kinase blockade
Source: Breast Cancer Res. 2017 Aug 4;19:90. doi: 10.1186/s13058-017-0882-x (PMC5545026; doi:10.1186/s13058-017-0882-x)
Supplement: Supplementary file 1 — The effect of gefitinib-FTY720 combination on proliferation of HCC1806 cells with stable IGFBP-3 knockdown. (A) Decreased IGFBP-3 production by two HCC1806 clones stably expressing shIGFBP-3 (shBP3) compared to two control clones expressing vector alone. (B) Cell proliferation data for combined shBP3 clones (red) compared to combined control clones (blue). Although shBP3 cells proliferate identically to control cells in the absence of treatment (solid symbols), treatment with the gefitinib-FTY720 combination (open symbols) is much more inhibitory to control cells than to cells with decreased IGFBP-3 expression. (C, D) Individual proliferation data for two HCC1806 control clones treated with gefitinib (2 μM), FTY720 (0.5 μM), or the combination. (E, F) Similar data for two HCC1806 shBP3 clones. Data are corrected for confluence at time zero, and are mean values ± SD at each time point for triplicate wells for each treatment. (PDF 420 kb) [file 13058_2017_882_MOESM1_ESM.pdf]

Supplementary Figure 1

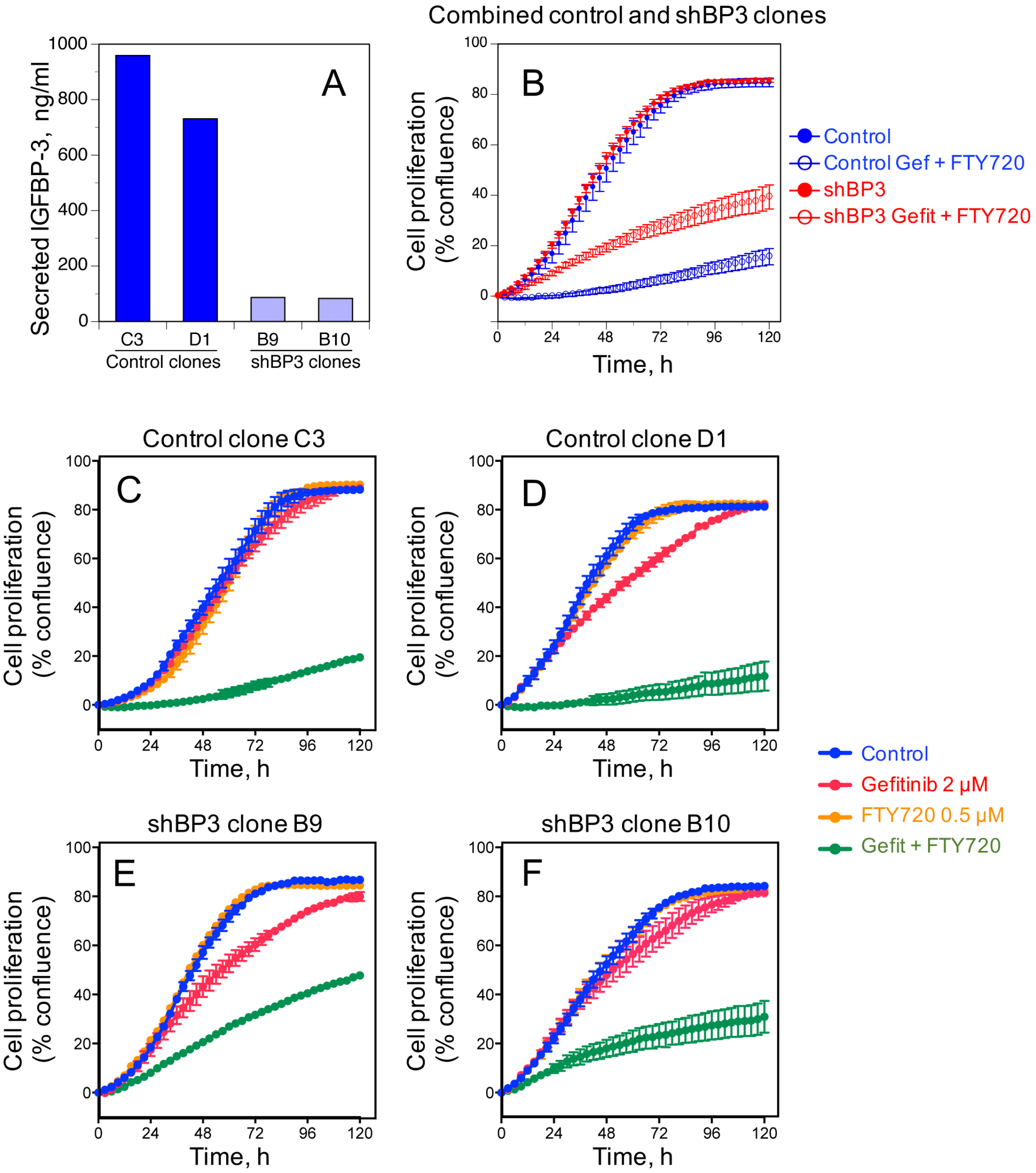

Supplementary Figure 1. The effect of gefitinib-FTY720 combination on proliferation of HCC1806 cells with stable IGFBP-3 knockdown.

A: Decreased IGFBP-3 production by two HCC1806 clones stably expressing shIGFBP-3 (shBP3) compared to two control clones expressing vector alone. B: Cell proliferation data for combined shBP3 clones (red) compared to combined control clones (blue). Although shBP3 cells proliferate identically to control cells in the absence of treatment (solid symbols), treatment with the gefitinib-FTY720 combination (open symbols) is much more inhibitory to control cells than to cells with decreased IGFBP-3 expression. C, D: Individual proliferation data for two HCC1806 control clones treated with gefitinib (2 µM), FTY720 (0.5 µM), or the combination. E, F: Similar data for two HCC1806 shBP3 clones. Data are corrected for confluence at time zero, and are mean values ± SD at each time point for triplicate wells for each treatment.
