# Supplementary material for: Inhibition of basal-like breast cancer growth by FTY720 in combination with epidermal growth factor receptor kinase blockade
Source: Breast Cancer Res. 2017 Aug 4;19:90. doi: 10.1186/s13058-017-0882-x (PMC5545026; doi:10.1186/s13058-017-0882-x)
Supplement: Supplementary file 2 — Relationship between IGFBP-3 mRNA and CD44 mRNA levels in HCC1806 clones with varying IGFBP-3 expression. Total RNA was extracted from duplicate cultures, reverse-transcribed and analyzed by qRT-PCR in duplicate as previously described [13]. Data points for each mRNA species were expressed relative to a single low-IGFBP-3 clone (B10) and were fitted using a four-parameter sigmoid logistic curve. (PDF 101 kb) [file 13058_2017_882_MOESM2_ESM.pdf]

Supplementary Figure 2

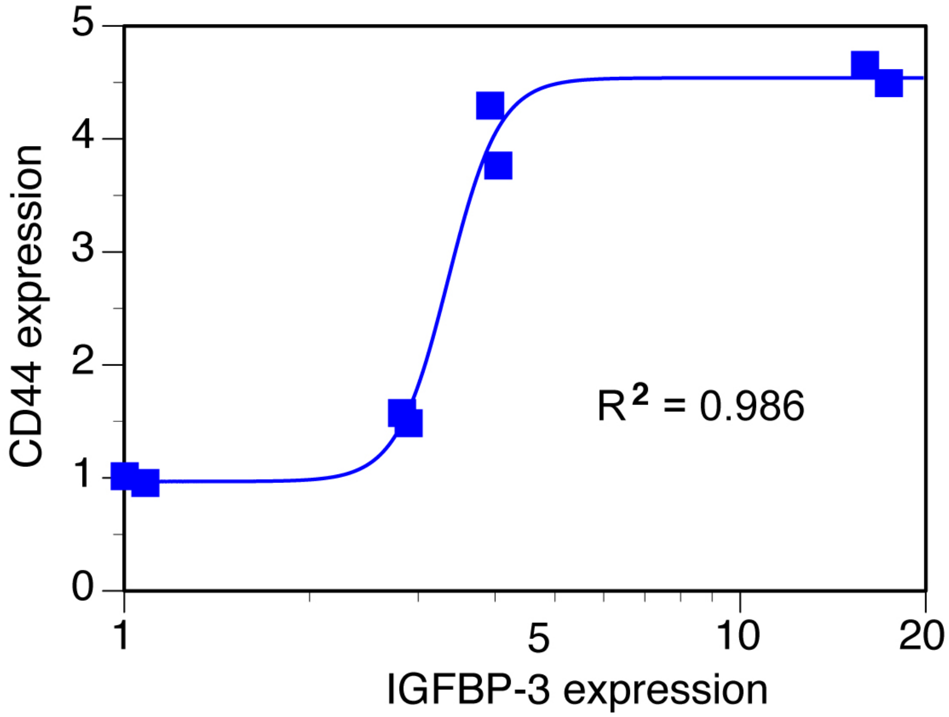

Supplementary Figure 2. Relationship between IGFBP-3 mRNA and CD44 mRNA levels in HCC1806 clones with varying IGFBP-3 expression. Total RNA was extracted from duplicate cultures, reverse-transcribed and analyzed by qRT-PCR in duplicate as previously described (13). Data points for each mRNA species were expressed relative to a single low-IGFBP-3 clone (B10) and were fitted using a 4-parameter sigmoid logistic curve.
