# Supplementary material for: Inhibition of basal-like breast cancer growth by FTY720 in combination with epidermal growth factor receptor kinase blockade
Source: Breast Cancer Res. 2017 Aug 4;19:90. doi: 10.1186/s13058-017-0882-x (PMC5545026; doi:10.1186/s13058-017-0882-x)
Supplement: Supplementary file 3 — CD3 levels in 4T1 tumors grown in wild-type BALB/c mice. Samples were from the experiment shown in Fig. 5d. (A) Western blot of three tissue lysates from each of the four treatment groups. α-Tubulin is shown as a loading control. (B) Quantitation of CD3 blots shown in Panel A, corrected for α-tubulin. Data are expressed as mean density values ± SEM. C. Immunohistochemistry of CD3 in three tumors in wild-type BALB/c mice from each of the four treatment groups, and one tumor in BALB/c nude mice from each treatment group as a negative control. (PDF 5088 kb) [file 13058_2017_882_MOESM3_ESM.pdf]

Supplementary Figure 3

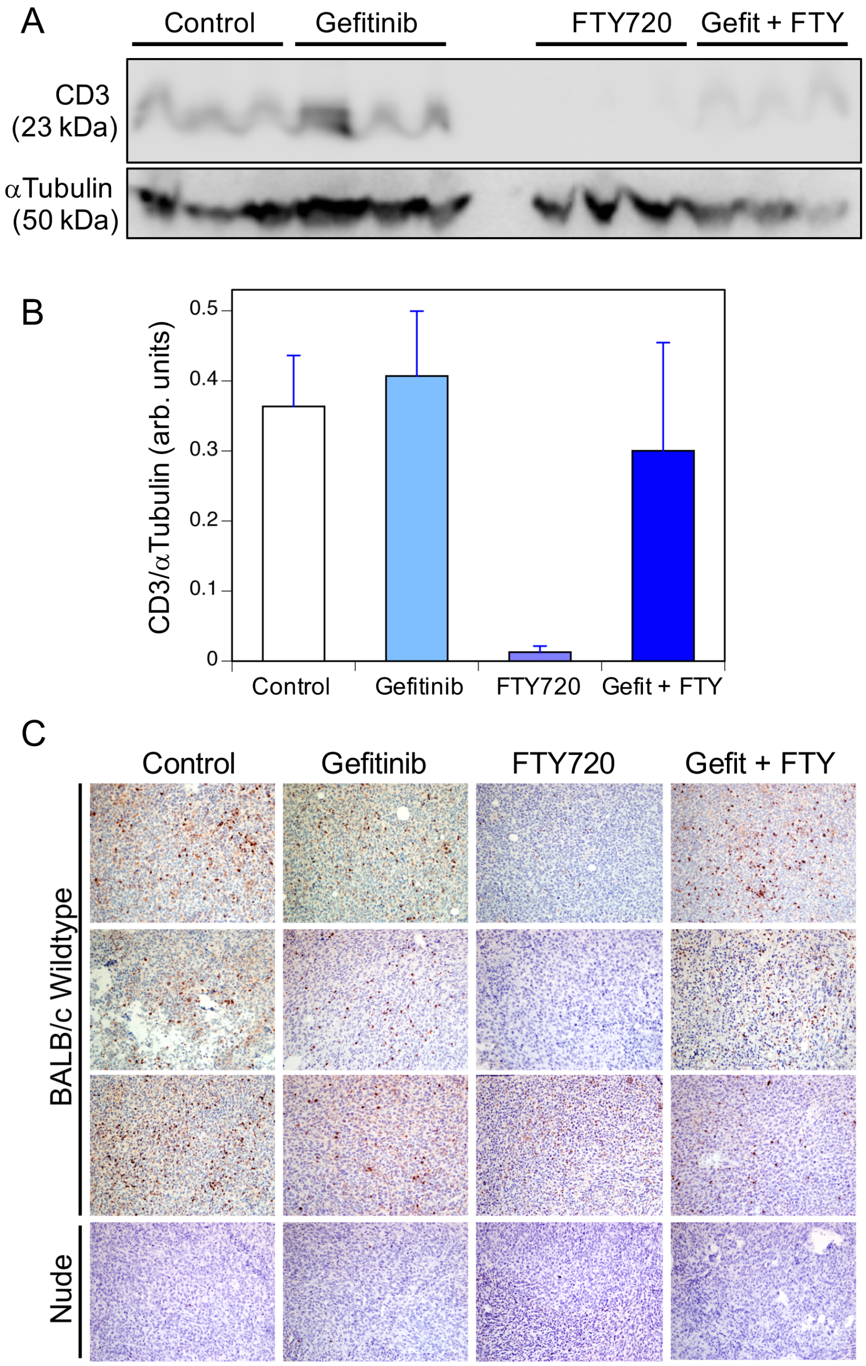

Supplementary Figure 3. CD3 levels in 4T1 tumors grown in wildtype BALB/c mice.

Samples were from the experiment shown in Figure 5D. A: Western blot of 3 tissue lysates from each of the 4 treatment groups. α-Tubulin is shown as a loading control. B: Quantitation of CD3 blots shown in Panel A, corrected for α-tubulin. Data are expressed as mean density values ± SEM. C. Immunohistochemistry of CD3 in 3 tumors in wildtype BALB/c mice from each of the 4 treatment groups, and one tumor in BALB/c nude mice from each treatment group as a negative control.
