# Supplementary material for: Inhibition of basal-like breast cancer growth by FTY720 in combination with epidermal growth factor receptor kinase blockade
Source: Breast Cancer Res. 2017 Aug 4;19:90. doi: 10.1186/s13058-017-0882-x (PMC5545026; doi:10.1186/s13058-017-0882-x)
Supplement: Supplementary file 4 — Correlations among immunohistochemical scores for cleaved caspase-3, Ki67, pEGFR and SphK1, in HCC1806 and MDA-MB-468 xenograft tumors. Data from the experiments are shown in Fig. 6. Correlations were assessed using Pearson’s correlation coefficient with two-tailed P values. (A, C, E) HCC1806 tumors. (A) cleaved caspase-3 vs. Ki67: n = 38, r = -0.602, P < 0.001. (C) Ki67 vs. pEGFR: n = 38, r = 0.576, P < 0.001. E: Ki67 vs. SphK1: n = 24, r = 0.462, P = 0.023. B, D, F: MDA-MB-468 tumors. (B) cleaved caspase-3 vs. Ki67: n = 25, r = -0.705, P < 0.001. D: Ki67 vs. pEGFR: n = 16, r = 0.726, P = 0.001. F: Ki67 vs. SphK1: n = 16, r = 0.614, P = 0.011. (PDF 308 kb) [file 13058_2017_882_MOESM4_ESM.pdf]

Supplementary Figure 4

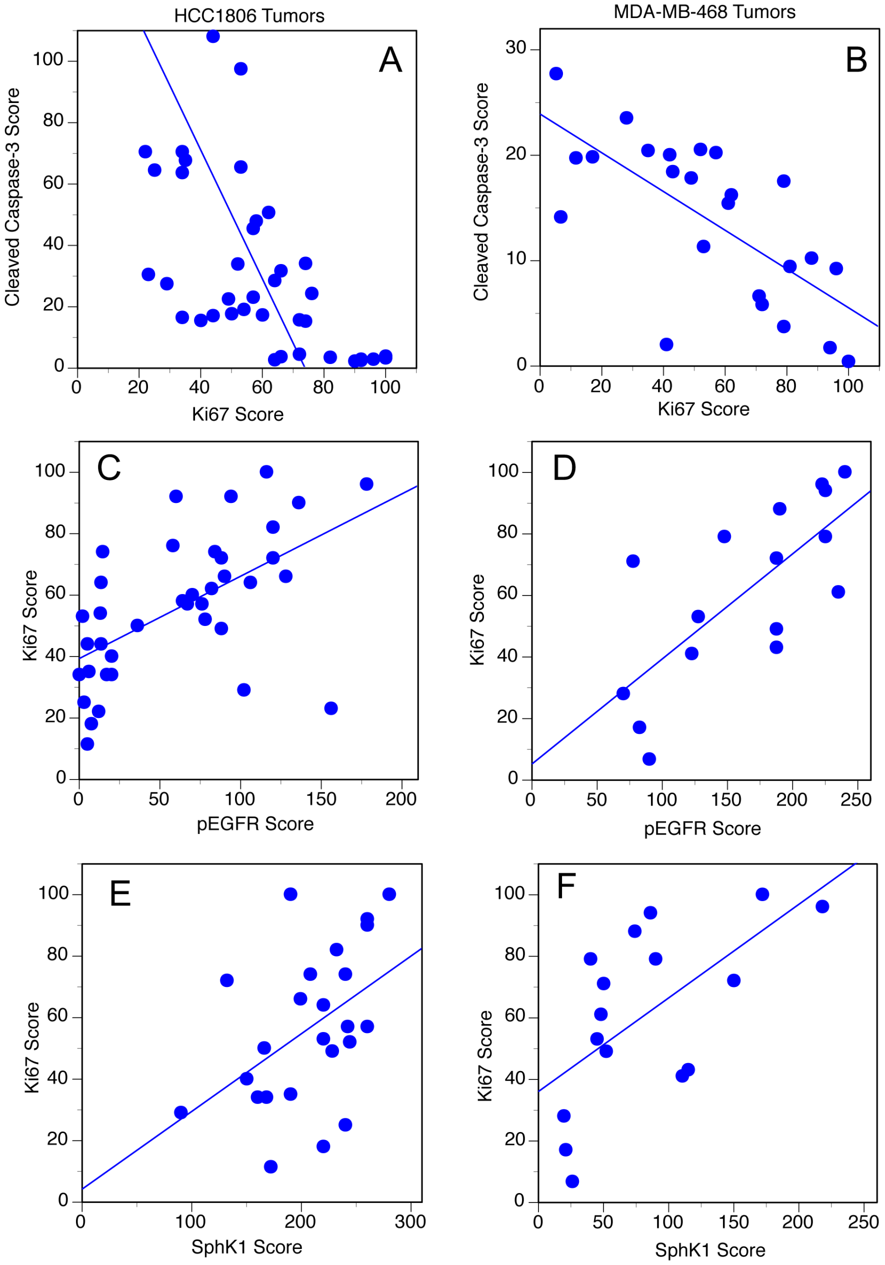

Supplementary Figure 4. Correlations among immunohistochemical scores for cleaved caspase-3, Ki67, pEGFR and SphK1, in HCC1806 and MDA-MB-468 xenograft tumors.
Data are from the experiments shown in Figure 6. Correlations were assessed using Pearson's correlation coefficient with 2-tailed P-values.

A, C, E: HCC1806 tumors. A: cleaved caspase-3 vs. Ki67: n = 38, r = -0.602, P<0.001. C: Ki67 vs. pEGFR: n = 38, r = 0.576, P<0.001. E: Ki67 vs. SphK1: n = 24, r = 0.462, P = 0.023.

B, D, F: MDA-MB-468 tumors. B: cleaved caspase-3 vs. Ki67: n = 25, r = -0.705, P<0.001. D: Ki67 vs. pEGFR: n = 16, r = 0.726, P = 0.001. F: Ki67 vs. SphK1: n = 16, r = 0.614, P = 0.011.
