# Supplementary material for: Inhibition of basal-like breast cancer growth by FTY720 in combination with epidermal growth factor receptor kinase blockade
Source: Breast Cancer Res. 2017 Aug 4;19:90. doi: 10.1186/s13058-017-0882-x (PMC5545026; doi:10.1186/s13058-017-0882-x)
Supplement: Supplementary file 5 — Immunohistochemical analysis of nuclear IGFBP-3 in HCC1806 tumors from control, gefitinib-treated (GEF), FTY720-treated (FTY), and combination-treated mice. (A) Tumors were stained for nuclear IGFBP-3, scored as described under Methods. Representative images from each treatment group (bar = 200 μm). (B) Summary data are mean values ± SEM (n = 6 per group), analyzed by one-way ANOVA followed by post hoc Tukey’s test. * P = 0.005, ** P < 0.001 compared to control; § P < 0.025 compared to combination. (PDF 691 kb) [file 13058_2017_882_MOESM5_ESM.pdf]

Supplementary Figure 5

A

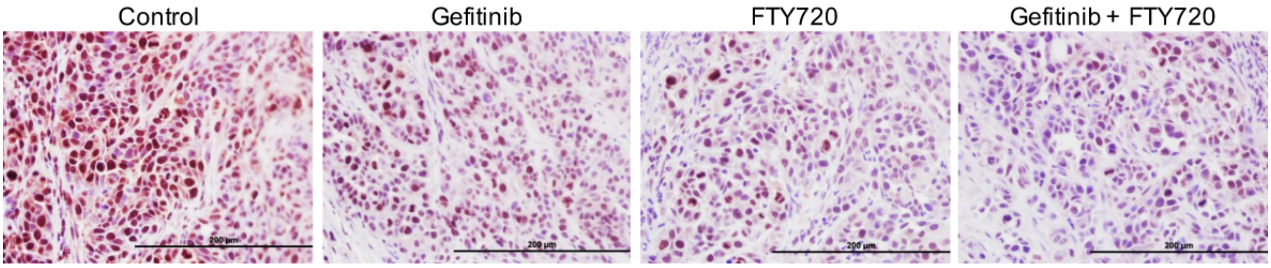

B

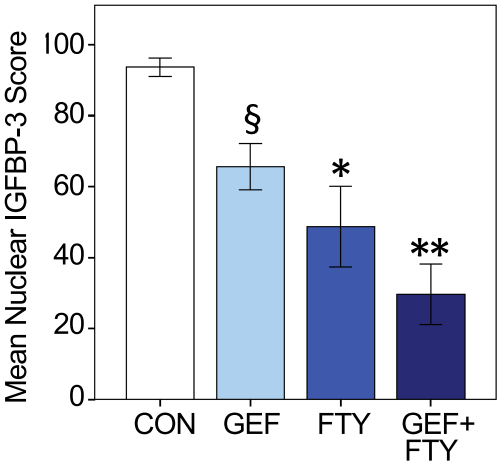

Supplementary Figure 5. Immunohistochemical analysis of nuclear IGFBP-3 in HCC1806 tumors from control, gefitinib-treated (GEF), FTY720-treated (FTY), and combination-treated mice.

A: Tumors were stained for nuclear IGFBP-3, scored as described under Methods. Representative images from each treatment group (Bar = 200 μm). B: Summary data are mean values ± SEM (n=6 per group), analyzed by 1-way ANOVA followed by post-hoc Tukey's test. *P=0.005, **P<0.001 compared to control; §P<0.025 compared to combination.
